# A focus on a complex abiotic tertiary structure

**DOI:** 10.1039/d5sc90011e

**Published:** 2025-01-30

**Authors:** Yulong Zhong, Bing Gong

**Affiliations:** a Department of Chemistry, University at Buffalo, The State University of New York Buffalo New York 14260 USA bgong@buffalo.edu

## Abstract

In contrast to the many well-defined helical secondary structures of foldamers reported thus far, examples of tertiary molecular structures of foldamers remain rare with the development of such folded structures being still in its infancy. While the direct design of foldamer tertiary structures still presents a daunting challenge, a realistic strategy for developing unimolecular tertiary structures of foldamers involves covalently linking the molecular components of known quaternary structures of foldamers that have been reported in recent years. Wang *et al.* (S. Wang, J. Sigl, L. Allmendinger, V. Maurizot and I. Huc, *Chem. Sci.*, 2025, **16**, 1136–1146, https://doi.org/10.1039/D4SC07336C), by starting from a *C*_3_-symmetrical, hydrogen-bonded homochiral parallel bundle of three aromatic helices, used rational principles and molecular modeling to convert the trimolecular object into a unimolecular helix-turn-helix-turn-helix tertiary structure that represents the most complex abiotic tertiary structure known to date.

## Abstract 2

This article highlights the recent report of Wang *et al.* (*Chem. Sci.*, 2025, https://doi.org/10.1039/D4SC07336C) on the successful creation of a complex abiotic tertiary structure based on the covalent connection of the molecular components of a known quaternary structure of helical foldamers.

## Graphical abstract

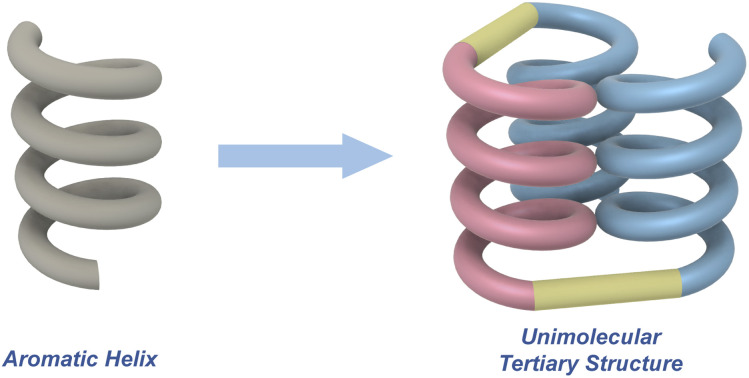

The folding and assembly of biomacromolecules have inspired the creation of various foldamers, artificial oligomers that fold into well-defined three-dimensional structures. Efforts over the past three decades have led to the creation of numerous foldamers that adopt well-defined secondary structures, most of which are helices.^1–6^ The availability of these discrete secondary structures has prompted efforts to design higher-level structures inspired by the tertiary and quaternary structures of proteins. Similar to those exhibited by peptides and proteins, including designed protein structures, the availability of tertiary and quaternary structures in foldamers should significantly expand the diversity of functions that can only be expressed at these structural levels, with applications in a range of fields including chemistry, biology, biomedical science, and materials science.

An early observation by Gellman *et al.* on the assembly of a β-peptide provided the first indication of a helical bundle, a quaternary structure of a foldamer.^7^ Indeed, in the solid state or in solution, for foldamers, many more examples of well-defined quaternary structures than tertiary structures have been observed. Most known foldamer quaternary structures consist of secondary structures, primarily various helices. An intriguing approach for developing tertiary structures of foldamers is to covalently link the secondary structural components of known quaternary structures. DeGrado *et al.* subsequently reported a two-helix bundle consisting of β-peptide helices linked *via* a disulfide bond.^8^ Other examples of foldamer tertiary structures include, but are not limited to, the peptoid helix bundles by Zuckermann and Dill,^9^ the β-peptide bundles reported by Schepartz,^10^ the sequence-guided backbone alteration by Horne,^11^ the AApeptide zipper by Cai,^12^ and the oliguria helix bundles by Guichard.^13^

Despite ongoing efforts, the development of protein-like tertiary structures with foldamers remains a significant challenge and is still in its infancy due to several reasons: (1) protein tertiary structures depend on a highly complex network of non-covalent interactions, whereas foldamers often lack the precise combination and tunability of such interactions, which are essential for achieving complex folding and assembly; (2) the synthesis of proteins is highly efficient, thanks to evolutionary advantages developed over millennia. In contrast, the synthesis of abiotic foldamers is limited by fewer available synthetic strategies and presents considerable challenges; (3) while protein tertiary structures are both dynamic and stable, it is still difficult to synthetically balance the rigidity and flexibility of foldamers.

The Huc group has been leading the development of tertiary structures for aromatic foldamers composed of aromatic rings in their main chain.^14^ Many aromatic foldamers, especially aromatic oligoamide foldamers,^4,6^ offer stably folded conformations, which enables the construction of higher-order structures with unique properties and functions that otherwise are unattainable with biomacromolecules.

The current work by Wang *et al.* involves the design of a unimolecular, three-helix bundle consisting of covalently linked aromatic helices that engage in hydrogen-bonding interactions (https://doi.org/10.1039/D4SC07336C).^15^ Starting from the known crystal structure of a *C*_3_-symmetrical homochiral, parallel bundle consisting of three aromatic helices that are held together by intermolecular hydrogen bonds,^14^ the authors, based on rational principles and molecular modeling, are able to convert the trimolecular object into a unimolecular helix-turn-helix-turn-helix tertiary structure, which is the most complex abiotic tertiary structure known to date.

To design a unimolecular three-helix bundle based on the crystal structure of the parallel trimer, the authors had to address two distinct challenges. The first was to organize the helices in space for them to be connected from the N-terminus of a helix to the C-terminus of an adjacent helix, which requires the realignment of one of the three helices, from being parallel to the other two helices to being antiparallel. This was achieved by inverting both the orientation and the handedness of one of the helices, a well-known operation adopted in the design of α-helical retro-inverso peptides,^16^ and also used by the Huc group to design the PM/MP helix-turn-helix tertiary fold.^17^ The resultant heterochiral trimolecular bundle was examined by molecular modeling, which confirmed the proper alignment of the helices and the placement of intermolecular hydrogen-bond patterns similar to those of the homochiral *C*_3_-symmetrical trimer. The other challenge was to design and synthesize linkers that covalently connect the three helices. Two distinct helix-helix connection strategies were envisaged, along with considering the rigidity of the linker to reduce the possibility of rearrangement and/or of introducing strain that may destabilize the tertiary structure, and examination using molecular modeling led to the design of two analogous turn units, T6f and T6r, that were synthesized and further examined (Fig. 1).

**Fig. 1:**
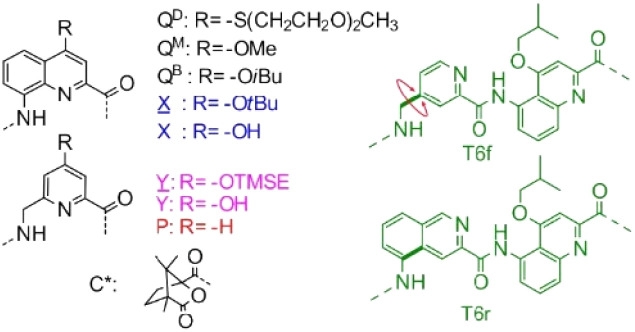
Structure of Q^D^, Q^B^, Q^M^, X, Y, P, T6f and T6r monomers as well as N-terminal chiral C* group. X̲ and Y̲ are the hydroxy-protected precursors of X and Y, respectively. TMSE = 2-trimethylsilylethyl. Reproduced from ref. 15 with permission from the Royal Society of Chemistry.

The design of the linkers was validated in helix-turn-helix models, first with shortened (three-residue) helices with sequences equivalent in length to Q_3_-turn-Q_3_, 2b, 3b and 4b, and then with oligomers 5b and 6b having the longer (eight-residue) helices equivalent in length to Q_8_-turn-Q_8_. Results based on solid-state structures and NMR studies demonstrate that T6f and T6r indeed mediate the helix-turn-helix arrangement, *i.e.*, U-shaped helix-turn-helix structures, desired to form the helix bundles, with the more rigid T6r being a better option to control conformation (Fig. 2).

**Fig. 2:**
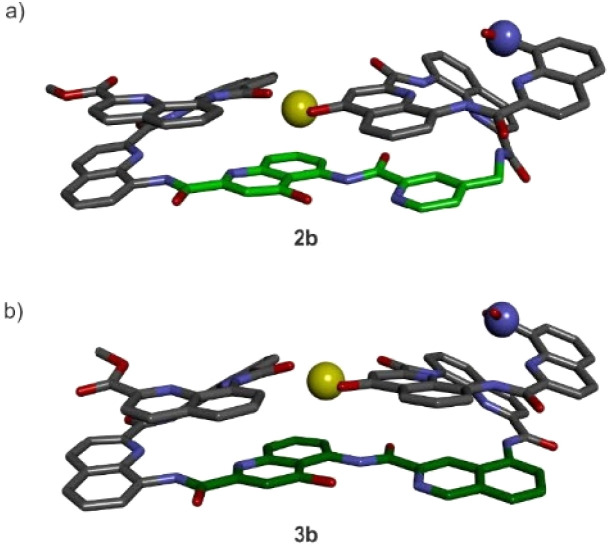
Crystal structures of 2b (a) and 3b (b). Reproduced from ref. 15 with permission from the Royal Society of Chemistry.

Oligomers 7b and 8b (Fig. 3a), designed to fold into unimolecular three-helix bundles, consisting of the same set of three helices connected by linkers T6f and T6r, respectively, were synthesized based on fragment condensation strategies that allowed the purification of the synthesized oligomers by recycling gel permeation chromatography (GPC) in chloroform.

**Fig. 3:**
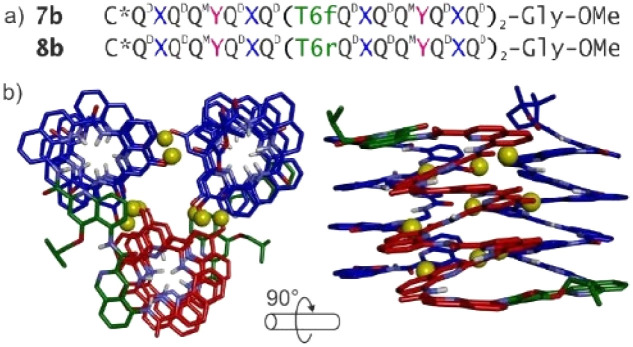
(a) Structural sequences of oligomer 7b and 8b. See Fig. 1 for the structures of residues Q^B^, Q^D^, Q^M^, and X, and linkers T6f and T6r. (b) Molecular model of 8b. The molecule is shown in stick representation. Nonpolar hydrogen atoms and side chains (except that of the T6r turn) have been removed for clarity. Hydroxy protons are shown as yellow balls. Reproduced from ref. 15 with permission from the Royal Society of Chemistry.

Oligomers 7b and 8b were extensively studied with NMR and CD spectroscopy. The obtained results indicate that oligomer 7b seems to exist as multiple species in both CDCl_3_ and CD_2_Cl_2_. In contrast, in CD_2_Cl_2_, data provided by ^1^H NMR, ^1^H,^15^N HSQC, DOSY, and CD spectra all point to 8b being a unimolecular tertiary fold with two helix handedness reversals held together by nine intramolecular hydrogen bonds. The well-defined folding of 8b confirms that T6r is a more effective linker than T6f and is consistent with the proposed molecular model (Fig. 3b). Interestingly, in CDCl_3_, oligomer 8b, instead of adopting the well-defined conformation observed in CD_2_Cl_2_, seems to exist as multiple species that remain to be identified. The reason for this solvent-dependent folding of 8b is not yet clear. The differing propensity of 7b and 8b to adopt well-defined conformations further demonstrates the crucial role of the linker in promoting the folding of these oligomers. The more flexible T6f turn in 7b failed to achieve defined folding, whereas the more rigid T6r turn in 8b provided sufficient stability for the folded structure.

The solvent-dependent folding of 8b suggests that the current design still has plenty of room for improvement. This may be achieved by enhancing the non-covalent interactions between the helical components, and by optimizing the design of the linker to further increase the stability of the overall folded structure.

In summary, a well-defined, unimolecular three-helix bundle based on oligomer 8b was successfully designed and obtained. The alignment of three helical components allows their connection from N-to-C termini, which was realized by inverting the orientation and the handedness of one of the three helices. Two different linkers, T6f and T6r, for connecting the helices, were designed based on molecular modeling and validated in helix-turn-helix models. Examining the folding of oligomers 7b and 8b, which differs by having linkers T6f and T6r, respectively, revealed the folding of 8b into an abiotic unimolecular three-helix bundle in CD_2_Cl_2_.

The current contribution presents a systematic and easy-to-follow approach based on rational principles, molecular modeling, and meticulous structural characterization. The developed method should be generally applicable for converting other known quaternary structures of foldamers into tertiary molecular structures by carefully considering the alignment of secondary structural components and by designing appropriate covalent linkers.

## Author contributions

B. G. wrote the manuscript with input and assistance from Y. L. Z.

## Conflicts of interest

There are no conflicts to declare.

## References

[cit1] Appella D. H., Christianson L. A., Karle I. L., Powell D. R., Gellman S. H. (1996). J. Am. Chem. Soc..

[cit2] Seebach D., Overhand M., Kühnle F. N. M., Martinoni B., Oberer L., Hommel U., Widmer H. (1996). Helv. Chim. Acta.

[cit3] Kirshenbaum K., Barron A. E., Goldsmith R. A., Armand P., Bradley E. K., Truong K. T. V., Dill K. A., Cohen F. E., Zuckermann R. N. (1998). Proc. Natl. Acad. Sci. U. S. A..

[cit4] Zhu J., Parra R. D., Zeng H., Skrzypczak-Jankun E., Zeng X. C., Gong B. (2000). J. Am. Chem. Soc..

[cit5] Semetey V., Rognan D., Hemmerlin C., Graff R., Briand J.-P., Marraud M., Guichard G. (2002). Angew. Chem., Int. Ed..

[cit6] Jiang H., Léger J.-M., Huc I. (2003). J. Am. Chem. Soc..

[cit7] Raguse T. L., Lai J. R., LePlae P. R., Gellman S. H. (2001). Org. Lett..

[cit8] Cheng R. P., DeGrado W. F. (2002). J. Am. Chem. Soc..

[cit9] Lee B.-C., Zuckermann R. N., Dill K. A. (2005). J. Am. Chem. Soc..

[cit10] Petersson E. J., Schepartz A. (2008). J. Am. Chem. Soc..

[cit11] Reinert Z. E., Lengyel G. A., Horne W. S. (2013). J. Am. Chem. Soc..

[cit12] Teng P., Niu Z., She F. Y., Zhou M., Sang P., Gray G. M., Verma G., Wojtas L., van der Vaart A., Ma S. Q., Cai J. F. (2018). J. Am. Chem. Soc..

[cit13] Pendem N., Nelli Y.-R., Cussol L., Didierjean C., Kauffmann B., Dolain C., Guichard G. (2023). Chem.–Eur. J..

[cit14] De S. M., Ch B., Granier T., Qi T., Maurizot V., Huc I. (2018). Nat. Chem..

[cit15] Wang S. H., Sigl J., Allmendinger L., Maurizot V., Huc I. (2025). Chem. Sci..

[cit16] Rai J. (2019). Chem. Biol. Drug Des..

[cit17] Mazzier D., De S., Wicher B., Maurizot V., Huc I. (2020). Angew. Chem., Int. Ed..

